# Sexual and reproductive health of newly-arrived asylum-seeking women: a cross-sectional survey in Finland

**DOI:** 10.1186/s12978-025-02012-2

**Published:** 2025-04-26

**Authors:** Satu Majlander, Tarja I. Kinnunen, Eero Lilja, Anu E. Castaneda, Natalia Skogberg, Päivikki Koponen

**Affiliations:** 1 Service System Unit, Finnish Institute for Health and Welfare, 00271 Helsinki, Finland; 2 Unit of Health Sciences, Faculty of Social Sciences, Tampere University, 33014 Tampere, Finland; 3 Welfare Epidemiology and Monitoring Unit, Finnish Institute for Health and Welfare, 00271 Helsinki, Finland

**Keywords:** Asylum-seeking women, Sexual and reproductive health, Nonresponse

## Abstract

**Background:**

Asylum-seeking women have an increased risk of sexually transmitted diseases, sexual and gender-based violence, unwanted pregnancies, maternal illness and death. This study examined sexual and reproductive health issues among asylum-seeking women in Finland in 2018. The acceptability of asking questions on sexual health was also evaluated.

**Methods:**

Data from the Asylum Seekers Health and Wellbeing Survey were used. Women aged 18–50 (n = 278) were included in the analysis and grouped to four categories based on their country of birth. Register information on sex, age group, country of birth, and place of residency was obtained from the Finnish Immigration Service and used in the calculations of analysis weights. Weighted percentages with 95% confidence intervals (Cl) were calculated for sexual activity, the use of contraceptives, female genital mutilation/cutting, pregnancies, previous births, miscarriages, induced abortions and menstrual health. The differences between the groups were compared using the chi-square test. The acceptability was examined based on nonresponse in each question about sexual health.

**Results:**

Among women from the other African countries (excl. North Africa), 21% (95% Cl 10.4–38.9%) had had six or more sexual partners within the past 12 months. Majority of women (62%, 95% CI 39.9–79.7%) from the ‘other countries’ and 51% (95% Cl 34.1–68.2%) from the other African countries had not used contraceptives during their latest intercourse. Female genital mutilation/cutting was reported by 30% (95% Cl 18.7–45.2%) of women from the other African countries. A total of 10% (95% Cl 6.6–13.9%) of all women and 25% (95% Cl 14.3–39.2%) of those from other African countries were pregnant at the time of study. Moreover, 35% (95% CI 25.5–46.0%) of the women from Russia and former Soviet Union had had at least one induced abortion. Nonresponse varied between 7 and 17%, being the highest in the questions about the gender of the sexual partner(s) and contraceptive use among women from Middle East and Africa.

**Conclusion:**

It is both acceptable and important to cover sexual and reproductive health when assessing the health of asylum-seeking women. The sensitivity of this topic must be considered when planning data collection.

**Supplementary Information:**

The online version contains supplementary material available at 10.1186/s12978-025-02012-2.

## Plain Language Summary

Asylum-seeking women face challenges related to sexual and reproductive health (SRH) and rights. They are at risk for sexually transmitted diseases, sexual and gender-based violence, unwanted pregnancies, and maternal illness and death. This study describes the sexual and reproductive healthcare needs of female asylum seekers in Finland with 2018 survey data. It also evaluates if it is acceptable to ask questions about SRH during health examination for asylum seekers. The study covers sexual behaviour, contraceptive use, female genital mutilation/cutting (FGM/C), pregnancies, births, and menstrual health among newly arrived asylum-seeking women in Finland. It also describes gaps in obtained data and nonresponse to sensitive questions.

Key findings:

- 21% of women from other African countries (excluding North Africa) had multiple sexual partners in the past year.
- Over 50% of women from other African countries and other countries did not use contraceptives during their latest intercourse.
- About 30% of women from other African countries had undergone FGM/C, with the mean age of five years.
- Nearly 50% of women from Russia and the Former Soviet Union had three or more pregnancies, and 35% had at least one abortion.
- 25% of women from other African countries were pregnant at the time of the study.
- Nonresponse was highest for questions about the gender of sexual partners and contraceptive use among women from the Middle East and other African countries.

It is important and acceptable to include SRH in health assessments for asylum-seeking women, but the sensitivity of the topic must be considered when planning interviews and examinations.

**Supplementary Information:**

The online version contains supplementary material available at 10.1186/s12978-025-02012-2.

## Background

Sexual and reproductive health (SRH) is a state of complete physical, emotional, mental, and social well-being in relation to sexuality and reproduction. It implies that people can have a satisfying and safe sex life, the capability to reproduce and the freedom to decide if, when, and how often to do so. Sexual and reproductive rights are fundamental rights [1, 2]. SRH services should include contraceptive services, maternal and newborn care, and prevention and treatment of sexually transmitted diseases [2], as well as safe and accessible abortion services [3]. The key targets of SRH counselling and services are to equally promote the comprehensive well-being of all people, prevent unwanted pregnancies and unsafe abortions as well as to prevent and treat sexually transmitted infections. Furthermore, healthcare professionals must be able to identify and support victims of gender-based violence [2, 4].

In different countries and cultures, there are several problems related to SRH and the associated women’s rights. For example, rights to contraception and access to SRH services can be restricted. Due to increased international migration, these issues are relevant questions worldwide. SRH issues have been essential for decades from the perspective of realizing women's rights, especially for women in vulnerable positions [5].

### Asylum-seeking women’s sexual and reproductive health

Asylum-seeking women tend to face more challenges than men relating to SRH and fundamental rights [1, 4]. However, for example asylum-seeking men belonging to sexual minorities can also be in a vulnerable position [6]. Asylum-seeking women have increased risk of sexually transmitted diseases, including HIV (human immunodeficiency virus), sexual and gender-based violence, unwanted pregnancies, maternal illness and death [2]. The reasons for these problems vary among asylum seekers coming to Finland: the situations in the country of origin, during the journey and in the country from which asylum is sought. For example, in countries at war, the SRH services are not available as they should [7]. In some cultures, issues related to religion limit the use of contraception or even access to all SHR services [8]. In addition, women seeking asylum are at risk of experiencing violence and exploitation during the asylum route [9, 10]. A previous study shows an association between a lack of housing and stable residence permits after migration and forced sex after migration [11].

One factor endangering women’s SRH is female genital mutilation/cutting (FGM/C). It is considered as a form of gender-based violence against girls and women [12]. In this context, FGM/C refers to all procedures that are performed for nonmedical reasons and involve partial or total removal of the external female genitalia or other injuries to the female genital organ. It has been estimated that more than 200 million girls and women alive today have been mutilated or cut in 30 countries in Africa, Asia, and the Middle East where FGM/C is most commonly practiced [13]. Immediate complications of FGM/C include excessive bleeding, infections, severe pain, shock, and even death. The long-term complications include urinary, vaginal, menstrual, and sexual problems as well as an increased risk of childbirth complications [14, 15].

In research and health promotion concerning the SRH of women seeking asylum, it is crucial to address their rights. Ensuring the rights to have children, not to want children, and to be a parent in a safe environment requires equitable and non-discriminatory health services. It is important to recognize that asylum-seeking women come from diverse backgrounds, each with unique experiences shaped by race, ethnicity, citizenship status, gender identity, and socioeconomic status. It is essential to understand how these intersecting identities affect their sexual- and reproductive health and rights [3].

We found only a few previous studies that have reported SHR among female asylum seekers [16, 17]. Most studies are based on data obtained from service providers and patient records, not directly from the asylum-seeking women themselves [16]. A study conducted among 80 asylum-seeking women in Switzerland utilized semi-structured interviews with healthcare providers and patient file data, revealing that limited access to contraception resulted in a 2.5 times higher rate of induced abortions among asylum-seeking women compared to the local population. Several SRH problems were observed, such as urogenital infections, lower-abdominal pain, spontaneous abortions, dysmenorrhea, and hypermenorrhoea/menorrhagia. Ten percent of the women had been raped, and 22.5% had experienced an unwanted pregnancy [17].

A study that assessed healthcare provision using semi-structured interviews with health and social care professionals (n = 9) working at the asylum centres in Switzerland showed that some women were probably raped during their journey and suffered from problems arising from this violence (including unwanted pregnancy and sexually transmitted diseases) [18].

### Asylum seeking women in Finland

In 2018, most people seeking asylum in Finland came were from Iraq, Russia, Turkey, Iran, Somalia, Syria, Afghanistan and Nigeria. A total of 30% of all applicants were women. Before 2018, asylum seekers came from these same countries, but the numbers have varied slightly every year. Most asylum-seekers coming to Finland have fled because of war or persecution. Reasons for being persecuted include e.g. religion and belonging to a sexual or gender minority [19–21]. There are some specific issues related to the SRH of asylum-seeking women coming to Finland. Most people seeking asylum in Finland come from countries where things like religion, values and traditions, gender equality, and the rights of sexual and gender minorities can affect the fact that issues related to sexual and reproductive health are considered sensitive.

A previous study conducted in Finland showed that repeated induced abortions are quite common among women of Russian origin, 32% of these women had had two or more induced abortions [22]. Some of the asylum-seekers come to Finland from countries where FGM/C still takes place. In Somalia, 99% of girls and women have undergone FGM/C [23], and the prevalence of FGM/C in Iraqi Kurdistan region is around 40% [24]. A previous study showed that 64% of Somali- and 32% of Kurdish-origin women living in Finland had undergone FGM/C [25]. Due to these risk factors, more research information is needed to develop the health examination offered to all asylum-seekers coming to Finland and to identify their needs for services.

### Feasibility and acceptability of studying sexual and reproductive health

Previous studies show that there might occur bias when answering questions related to SRH and experiences of violence. A previous study reported several factors affecting the data collection among Rohingya refugees in Bangladesh: sensitivity regarding SRH among participants, identifying appropriate sampling strategies, and addressing community trust issues [26]. Women might wish to conceal their experiences of violence [27]. Additionally, although women experience pain during menstruation (for example due to FGM/C), they might not want talk about these problems [26]. In a study in which the questionnaire was designed following a consultative process with the asylum-seeking population in a migrant camp in Greece, the overall response rate was over 80%. However, 13.4% of the respondents declined to answer the questions about sexual and gender-based violence [9].

As questions on SRH are often considered sensitive and may be liable to item nonresponse or bias, the research fieldwork personnel have a significant role in contacting the participants and inviting them to participate in the study, as well as ensuring a feeling of trust during the data collection [16, 28, 29].

Structured survey questionnaires should be translated into different languages whenever feasible, and it is important to use professional interpreters who are competent in choosing terms which the respondents understand similarly [29]. A study, in which 29 providers of refugee services for migrants from Burma in Australia were interviewed, showed that there is often a language barrier between the migrant and the healthcare professionals. In a study situation, a competent interpreter is needed for both parties to understand the questions and response options similarly. It also needs evaluating how the interpreter may impact the communication. Healthcare providers described several techniques and strategies to optimize communication during interpreter-mediated SRH consultations with refugee patients, e.g. gender matching of the interpreter, on-site interpreters instead of interpreting by telephone, and professionals rather than family members as interpreters [30].

### Study purpose and aims

There is very little research information available on asylum-seeking women’s SRH in Finland and most other European countries. Information is needed for the development of services and for supporting the work of healthcare professionals. The purpose of this study is to identify sexual and reproductive healthcare needs among female asylum seekers and to evaluate the acceptability of asking questions regarding SRH issues in the health examinations of asylum seekers in Finland using data collected in 2018. Specifically, this study will examine, sexual behaviour, contraception, female genital mutilation/cutting, pregnancies (including miscarriages and abortions), births and menstrual health among newly arrived asylum-seeking women in Finland. The results are presented alongside with nonresponse in these sensitive questions and gaps in information obtained. Information on acceptability was also needed for implementing the study results to the development of health examinations offered to asylum seekers in Finland in the future.

## Materials and methods

### The study population

The cross-sectional Asylum Seekers Health and Wellbeing Survey (TERTTU) was conducted by the Finnish Institute for Health and Welfare in 2018 [31]. The survey was conducted in collaboration with the Finnish Immigration Service and the reception centres. At the beginning of the asylum application process, a substantial majority of adults and families are directed to the transit reception centres where they wait for the asylum interview. After this, persons seeking asylum move to the reception centres to wait for an asylum decision. At the time of the TERTTU survey, there were altogether 49 reception centres and four transit reception centres. The transit centres were geographically dispersed across the country in the cities of Helsinki, Turku, Joutseno, and Oulu. Most asylum seekers (94%) who took part in the TERTTU Survey resided in one of these four transit centres at the point of data collection. However, a smaller part of the participants had already moved to other longer-term reception centres. In addition, some resided in private accommodation.

In the TERTTU survey, the aim was to invite to the survey all newly-arrived asylum seekers (both adults and children, including unaccompanied children) who had applied for asylum in Finland during the period between the 19th of February and the 30th of December 2018. The register data on newly-arrived asylum seekers were transferred on a weekly basis to the research team during the data collection phase. The electronic database is maintained by the Finnish Immigration Services, and it contains data on all asylum seekers in Finland. Asylum seekers were invited to participate in the study approximately two weeks after the registration of their asylum application in Finland, so that the research personnel could reach them before they moved to wait for the processing of their asylum application elsewhere in Finland.

**Fig. 1:**
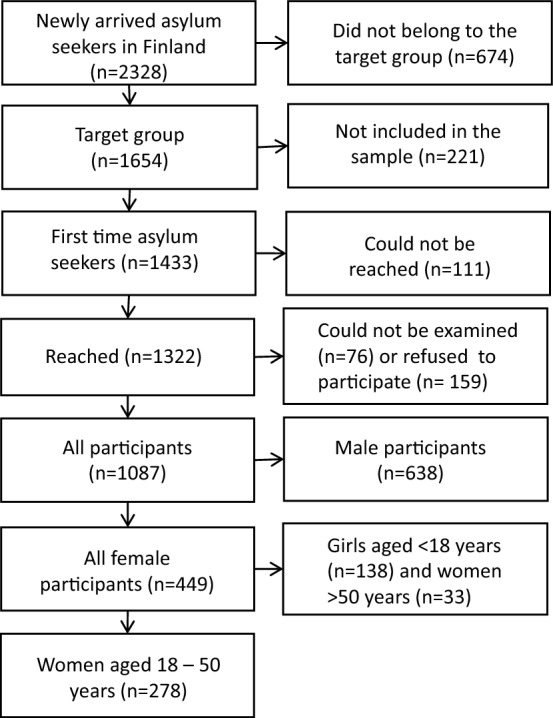
Flow chart of the selection of the study participants

In total, 2328 newly arrived asylum seekers were registered during the study period (Fig. 1). Of them, 674 fulfilled at least one of the following exclusion criteria indicating that the person would not belong to the key target groups for developing health examinations for newly arrived asylum seekers in Finland or that it would not be feasible to invite them to participate in the study: residence in a detention centre, previous applications for asylum in other countries and transfer to Finland based on international agreements (EU internal transfer), having been returned to Finland in accordance with the EU Dublin Regulation, previous application for residence permit in Finland, or health reasons reported by reception centre staff that would prevent participating in the study.

Additionally, 221 persons were not contacted due to technical problems resulting in a study sample of 1433. Of the persons in the sample, 784 adults and 303 under 18-year-old children took part in the TERTTU survey. Overall participation rate was excellent both among men (76%) and women (76%). In this study, only women aged 18–50 years (n = 278) were included in the analysis. The research methods of the TERTTU survey have been described in more detail in a previous publication [31].

### The data collection

The data collection of the TERTTU survey was implemented by eight trained multilingual research nurses, six of whom were women. Research nurses were employed by the research organisation. The research nurses conducted the interviews in all those places where the asylum seekers were accommodated. Whenever possible, the women were interviewed by a female research nurse. All except one had a degree in healthcare. The participant had the opportunity to request a different research nurse.

The research nurses commanded the most commonly spoken languages among the participants. The language was either participant’s mother language or a language that both could speak well enough [31]. In addition to having a good command of Finnish, the team of research nurses spoke English, Arabic, Somali, Persian, Dari, Sorani dialect of Kurdish, Urdu, Russian, Portuguese, and French. If necessary, it was possible to use a professional interpreter in the interviews. A total of 39% of the participants needed an interpreter, and these interviews were mostly conducted by telephone, allowing the anonymity of the participants.

The survey material given to the participants (including information leaflets and consent forms) was translated into the most common languages spoken by the asylum seekers arriving at the beginning of 2018, that is into English, Arabic, Somali, Persian, Sorani dialect of Kurdish, and Russian.

The TERTTU survey consisted of a standardized face-to-face interview and a health examination that followed a standardised protocol [31]. The structured interview included several questions about the participants’ background, health, and wellbeing including questions on topics such as asylum-seeking journey, socio-economic status, health problems and symptoms, traumatic experiences, and SRH. The participants answered the questions mainly in a face-to-face interview. Answering was voluntary, but the research nurses were trained to explain why these questions were included in the study.

An opportunity to complete self-filled structured survey questionnaires was offered for some sections of the survey (e.g. sections on SRH). Answering the structured questionnaire independently was considered as the most feasible way to cover questions on SRH. If necessary, it was possible to fill the questionnaire with the help of research nurse/assistant. A total of 37% of those who were offered the self-administered questionnaire, needed this help to answer (e.g., due to limited or no reading or writing skills).

### Variables

#### Participants’ background

Information on sex, year of birth, and country of birth of the participant was obtained from the electronic database on all asylum seekers maintained by the Finnish Immigration Services. The sex of a person was categorized as man or woman [31]. Information about a person’s age was calculated from the year of birth to the time of study and categorized as 18–29, 30–38, or 39–50 years. In this study, participants were grouped into four regions of origin based on their country of birth: (1) Russia or the former Soviet Union (FSU), (2) the Middle East and North Africa (MENA), (3) other African countries (excluding North Africa), and (4) other countries. The MENA group included mainly citizens of Turkey, Iran, and Iraq, while the other African countries group included mainly citizens of Somalia, Nigeria, Angola, and Cameroon. The countries of birth in the other countries group included Nicaragua, Albania, Bangladesh, India, Cuba, Kosovo and Sri Lanka. Other background variables (education, reading and writing skills, language skills, family situation) were based on self-reported information, and their classification can be found in Appendix 1.

### Sexual health

When the questions regarding sexual health were first introduced in the questionnaire, there was a note explaining that the questions could be used to identify risk behaviour connected with certain diseases and to recognize possible needs for care, protection, support, or help. The participants were informed of the fact that all the answers would be treated confidentially. There were explanations offered before the questions, explaining e.g. that in Finland it is forbidden to discriminate against anyone based on sexuality or sexual behaviour, and giving information on topics such as contraception (Appendix 1). The questions about sexual activity addressed the degree of sexual activity, the number of sexual partners, and the gender of the sexual partner(s) and were limited to the last 12 months. In addition, the participants were asked about the use of contraceptive methods and need for contraceptives. The questionnaire also included a question about FGM/C. A definition of FGM/C was given alongside the question about FGM/C.

### Reproductive health

The questions about reproductive health addressed the respondent’s current pregnancy status, the duration of pregnancy in weeks (if applicable), the numbers of previous pregnancies, births, miscarriages and induced abortions, and problems relating to childbirth (e.g. a difficult tear stitched by a doctor/stitched under sedation, C-section or other procedure, and more than usually painful labour or prolonged labour), and menstrual health (see Appendix 1).

### Acceptability of addressing questions on sexual and reproductive health

In the questions about sexual health, it was possible to choose the option ‘I do not want to answer’, as these questions were considered to be particularly sensitive. Suggestion to include this answer option for the questions which were considered as most sensitive was made by the multilingual fieldwork personnel during their training. The acceptability of the sexual health questions was examined by how the participants answered the questions. Answer ‘I do not want to answer’ or if the respondent did not choose any of the answer options were considered as nonresponse indicating low acceptability. It was not possible to choose the option ‘I do not want to answer’ for questions on FGM/C.

Based on discussions with research nurses, the questions about reproductive health were not considered as difficult to address or answer as questions about sexual health. Therefore, information on whether the respondent did not want to answer was not available (see Appendix 1).

### Statistical analyses

In this study, all analyses were conducted using the SAS 9.4 statistical software. Analysis weights were applied in order to reduce nonresponse bias. The weights were calculated using the inverse probability method. In this method, the probability of participation was estimated for each participant using register information available for survey participants and non-participants. The estimated probabilities were then inversed and thus used to correct the results to represent the entire asylum seeker population in Finland in 2018. Register information on sex, age group, country of birth, and residency location (= reception centres or private accommodation), obtained from the Finnish Immigration Service was used in the calculation of the weights. The estimation was performed using the *predict* function in the R package random Forest [32].

Weighted percentages for sexual activity, use of contraceptives, FGM/C, pregnancies, previous births, miscarriages, induced abortions and menstrual health as well as their 95% confidence intervals (CI) for binary and multinominal variables were calculated. The confidence intervals were calculated using a logit transformation that is suited for proportions that are near zero. The differences between the country groups were assessed using the chi-square test.

Frequencies and unweighted percentages were reported by country group for the item non-response results. The weights were not applied in these analyses, as the results were solely used to assess the acceptability of the questions.

## Results

### Background of the study participants

Of the women aged 18–50 included in the study (n = 278), all answered at least some of the questions concerning SRH. The participating women had come to Finland from Russia and FSU (30%), MENA (43%), other African countries (17%), and other countries (9%). Most of the women were 18–38 years old at the time of study and had received at least high school or vocational training (Table 1). The proportion of women who spoke only their mother tongue was 34% among women from Russia and FSU and 32% among women from other African countries.

**Table 1:** Background of the study participants, weighted percentages (95% confidence intervals)

	Russia and FSU	MENA^a^	Other African countries	Other countries	Total
	n = 85 % (95% Cl)	n = 119 % (95% Cl)	n = 47 % (95% Cl)	n = 27 % (95% Cl)	n = 278 % (95% Cl)
**Age**					
18–29 years	29.3 (20.7–39.8)	31.0 (23.2–40.0)	46.5 (32.3–61.2)	27.6 (12.1–51.4)	32.9 (27.4–38.8)
30–38 years	43.6 (33.5–54.2)	48.8 (39.8–58.0)	36.2 (23.8–50.8)	49.7 (30.7–68.9)	45.2 (39.3–51.3)
39–50 years	27.1 (18.8–37.5)	20.2 (13.8–28.6)	17.3 (7.3–35.8)	22.7 (10.0–43.6)	21.9 (17.2–27.5)
**Education**					
No education or only elementary school education	4.9 (1.8–12.2)	21.2 (14.6–29.8)	45.4 (31.2–60.4)	16.5 (5.9–38.2)	20.2 (15.7–25.6)
High school or vocational training	59.8 (49.1–69.6)	28.4 (20.8–37.5)	35.1 (21.7–51.4)	42.2(24.3–62.4)	40.1 (34.3–46.2)
University degree	35.3 (26.0–46.0)	50.4 (41.2–59.6)	19.5 (10.7–33.0)	41.3 (23.9–61.2)	39.7 (34.0–45.7)
**Language skills**					
Speaks only her mother tongue	34.0 (24.6–44.7)	27.4 (20.1–36.1)	31.9 (19.4–47.7)	22.7 (10.0–43.6)	29.5 (24.3–35.3)
**Reading skills**					
Can at most read simple texts	2.4 (0.6–8.9)	12.5 (7.6–19.9)	32.3 (20.6–46.8)	13.3 (4.0–36.0)	13.2 (9.6–17.8)
**Writing skills**					
Can at most write simple texts	3.5 (1.1–10.3)	12.5 (7.6–19.9)	34.2 (22.2–48.7)	13.3 (4.0–36.0)	13.8 (10.2–18.5)
**Family**					
Spouse in Finland or abroad	81.1 (71.4–88.1)	75.9 (66.7–83.2)	25.2 (13.7–41.7)	50.6 (31.4–69.7)	66.0 (59.9–71.6)
No spouse	18.9 (12.0–28.7)	24.1 (16.8–33.4)	74.8 (58.3–86.3)	49.3 (30.3–68.6)	34.0 (28.4–40.2)

^a^ The Middle East and North Africa

Women from the other African countries differed from the others: Of them, 45% had no education or had primary school education as their highest education, and 32–34% could only read and write simple texts. In addition, 75% reported that they do not have a spouse or that their spouse had died.

### Sexual health of the study participants

Among all groups, 65–82% had had sex with at least one person within the past 12 months. Among women in the other African countries group, 44% had had more than one sexual partner, and 21% (10.4–38.9%) had had six or more sexual partners within the past 12 months (Table 2). Among women in the other countries groups there were only one or two persons who had had more than one sexual partner, preventing statistical analysis. Among women in the other African countries group there were four persons who had had sexual partner of the same sex, in the other groups there were none.

**Table 2:** Sexual health among asylum-seeking women aged 18–50 years, weighted percentages (95% confidence intervals)

	Russia and FSU n = 85 % (95% Cl)^b^	MENA^a^ n = 118 % (95% Cl)^b^	Other African countries n = 47 % (95% Cl)^b^	Other countries n = 27 % (95% Cl)^b^	*p*-value^e^	Total n = 277 % (95% Cl)^b^
**Sexual activity**						
At least one sex partner within the past 12 months	82.2 (72.2–89.1)	65.4 (55.7–74.0)	75.4 (60.0–86.3)	70.2 (48.3–85.6)	0.131	72.4 (66.3–77.7)
> 1 sex partners within the past 12 months^d^	NA^c^	NA^c^	43.8 (26.8–62.4)	NA^c^	NA^c^	9.17 (5.7–14.5)
**Use of contraceptive(s)**						
Did not use any contraceptives during the latest intercourse^d^	44.5 (33.0–56.6)	43.2 (32.7.–54.3)	51.3 (34.1–68.2)	61.7 (39.9–79.7)	0.426	47.3. (40.3–54.3)
Need for contraception	16.8 (10.1–26.8)	26.9 (19.2–36.3)	NA^c^	24.2 (10.7–46.0)	0.293	19.8 (15.2–25.3)
**Female genital mutilation/cutting**						
Undergone FGM/C	NA^c^	6.6 (3.3–2.6)	30.4 (18.7–45.2)	NA^c^	** < 0.001**	8.4 (5.6–12.3)

^a^ The Middle East and North Africa

^b^ % of those who responded to the question, those who did not wish to answer or skipped the question were excluded

^c^ NA: Not available as too few observations (n =  < 5) for statistical analysis

^d^ % of those who had had intercourse within the past 12 months

^e^ calculated with the chi-square test, a bolded p-value < 0.05 was considered statistically significant

Over half of the women in the other African countries group and the other countries groups had not used any contraceptive methods during their latest intercourse. Among women in the Russia and FSU group and the MENA group, the corresponding proportion was 43–45%. More than a quarter of women in the MENA group, 24% of women in the other countries group and 17% of women in the Russia and FSU group reported that they need contraception. About 30% of women in the other African countries group had undergone FGM/C, and the mean age at the time of FGM/C was five years.

### Reproductive health of the study participants

A tenth of all participants were pregnant at the time of study, while the corresponding proportion was a quarter among women in the other African countries group (Table 3). Regarding weeks of pregnancy, there was one missing answer, one woman was pregnant, but the weeks of pregnancy were not known, and 11 women did not know if they were pregnant. Among the pregnant women who knew the duration of their pregnancy, 40% of pregnancies had lasted 20 weeks or more.

**Table 3:** Reproductive health among asylum-seeking women aged 18–50, weighted percentages (95% confidence intervals)

	Russia and FSU n = 85 % (95% Cl)^b^	MENA^a^ n = 118 % (95% Cl)^b^		Other African countries n = 47 % (95% Cl)^b^	Other n = 27 % (95% Cl)^b^	*p*-value^e^	Total n = 277 % (95% Cl)^b^
**Pregnant**							
Pregnant at the time of study	6.3 (2.7–14.2)		7.5 (3.7–14.6)	24.7 (14.3–39.2)	NA^c^	** < 0.001**	9.7 (6.6–13.9)
**Previous pregnancies**							
0	22.4 (14.6–32.8)		25.7 (18.1–35.1)	42.1 (27.8–57.8)	38.1 (20.2–60.0)	**0.048**	29.0 (23.5–35.2)
1–2	28.7 (19.9–39.4)		43.0 (33.8–52.5)	30.2 (18.7–45.1)	36.2 (19.7–56.8)		35.9 (30.2–42.0)
3 or more	48.9 (38.2–59.7)		31.4 (23.4–40.8)	27.7 (15.6–44.3)	25.7 (12.8–44.7)		35.1 (29.4–41.2)
**Previous births**							
0	29.9 (20.9–40.7)		31.5 (23.2–41.0)	48.1 (33.2–63.4)	48.7 (29.3–68.5)	0.231	35.8 (30.0–42.2)
1–2	47.5 (36.9–58.4)		48.8 (39.4–58.2)	39.0 (25.4–54.6)	41.7 (24.3–61.4)		45.9 (39.8–52.2)
3 or more	22.6 (14.7–33.0)		19.8 (13.2–28.5)	12.8 (5.1–28.7)	NA^c^		18.2 (13.9–23.5)
Difficulties in childbirth^d^	49.1(36.5–61.8)		50.6 (38.9–62.3)	36.4 (19.3–57.9)	68.8 (43.4–86.3)	0.278	49.8 (42.1–57.5)
**Miscarriages**							
0	75.1 (64.5–83.3)		80.1 (71.9–86.4)	75.1 (59.4–86.1)	77.5 (58.8–89.3)	0.844	77.5 (72.1–82.2)
≥ 1	24.9 (16.7–35.5)		19.9 (13.6–28.1)	24.9 (13.9–40.6)	22.5 (10.7–41.2)		22.5 (17.8–27.9)
**Induced abortions**							
0	64.9 (54.0–74.5)		90.9 (83.7–95.1)	87.2 (71.3–94.9)	76.6 (55.2–89.7)	** < 0.001**	81.3 (75.9–85.7)
≥ 1	35.1 (25.5–46.0)		9.1 (4.9–16.3)	12.8 (5.1–28.7)	23.4 (10.3–44.8)		18.7 (14.3–24.1)
**Menstrual health**							
Bothersome menstrual pain	31.2 (21.9–42.3)		41.7 (32.6–51.3)	27.9 (16.5–43.1)	24.3 (10.1–47.8)	0.230	34.5 (28.7–40.7)
Heavy periods	25.9 (17.4–36.7)		27.2 (19.7–36.4)	23.6 (13.3–38.4)	27.6 (12.5–50.4)	0.976	26.3 (21.1–32.2)
Irregular periods	18.4 (11.2–28.6)		39.9 (30.8–49.7)	40.2 (25.5–56.8)	38.1 (20.2–60.0)	0.032	33.8 (27.9–40.2)

^a^ The Middle East and North Africa

^b^ % of those who responded to each question, those who didn’t want to answer were excluded

^c^ NA: Not available as too few observations (n =  < 5) for statistical analysis

^d^ % of those who have given birth

^e^ calculated with the chi-square test, a bolded p-value < 0.05 was considered statistically significant

Of the women in the MENA group, 43% had had 1–2 previous pregnancies, whereas almost half (49%) of the women in the Russia and FSU group had had three or more pregnancies (including miscarriages and induced abortions). Of the women in the Russia and FSU group, almost a quarter had had three or more previous births. Difficulties during childbirth were reported by 70% of women in the other countries group and about a half of women in the Russia and FSU group (49%) and the MENA group (51%).

The proportion of women who had experienced miscarriages was between 20 and 25% in all groups. In almost all groups, there was at least one woman who had experienced repeated miscarriages, but there were too few observations for statistical analysis. Induced abortions were the most common among the women in the Russia and FSU group (35% had had one or more). A few women (2 or 3 persons) in different groups had experienced more than two induced abortions.

Of the women in the MENA group, 42% reported bothersome menstrual pain and about a quarter (24% to 28%) of all the women reported heavy periods. In addition, 40% of women in the MENA and other African countries groups reported irregular periods.

### Acceptability of addressing questions about sexual health

Almost a tenth (7%) skipped the question or did not wish to answer the question regarding sexual activity (Table 4). In addition, 19% of women in the other African countries group skipped the question or did not wish to answer the question about the number of sexual partners.

**Table 4:** Women who skipped the question or did not wish to answer^a^

	Russia and FSU n = 71–82^c^ % (n)	MENA^b^ n = 84–114^c^ % (n)	Other African countries n = 37–45^c^ % (n)	Other countries n = 21–27^c^ % (n)	Total n = 213–268^c^ % (n)
Sexual activity	7.3 (6)	8.8 (10)	4.4 (2)	3.7 (1)	7.1 (19)
Number of sexual partners	4.2 (3)	10.7 (9)	18.9 (7)	14.2 (3)	10.3 (22)
Gender of the sexual partner(s)	14.1 (10)	21.4 (18)	10.8 (4)	19.5 (4)	16.9 (36)
Use of Contraceptive(s)	12.1 (9)	18.0 (18)	21.4 (9)	8.0 (2)	15.8 (38)

^a^ Unweighted percentages and number of non-respondents

^b^ The Middle East and North Africa

^c^ Varies by item

The proportion of those who skipped the question or did not wish to answer the question about the gender of the sexual partner(s) was 17% among all participants, 21% among women in the MENA group and 14% among women in the Russia and FSU group. A total of 16% skipped the question or did not wish to answer the question about the use of contraception, and this was most common (21%) among women in the other African countries group.

## Discussion

### Sexual health

There are special issues relating to the SRH of asylum-seeking women depending on their background, the events during the asylum route, and the woman's current situation [5]. In this study a fifth of the women in the other African countries group had had multiple sexual partners in the past 12 months and only 65% of the MENA group reported that they had had sex with at least one person within the past 12 months.

Talking about sexuality can be difficult due to the shame or stigma associated with it. In some cultures, for example, premarital sex is not allowed, and sexual minorities may be discriminated [33, 34]. However, asking questions about sexual activity is important because it provides an opportunity to address sexual health issues and offer sexual counselling. Especially among female asylum seekers, changing sexual partners and having multiple partners might indicate that the woman has been a victim of human trafficking or exploitation [10].

In our study, over half of the women in the other African countries group and in the other countries group had not used any contraceptive methods during their latest intercourse. The corresponding proportion was 45% among women of Russian and FSU group. All of them didn’t consider that they need contraception, as only about a fifth of the women answered that they need contraception. According to 2021 World Bank data, 33% of African women (aged 15–49) use contraception, with significant variation between countries: 67% in Zimbabwe, 4% in South Sudan, and 7% in Somalia [35]. Additionally, 2020 statistics indicate that 41% of women of Russian background used modern contraception [36].

Religion and cultural factors may influence on what contraception a woman can or is willing to use [8]. It is possible that some women in this study did not need contraception because they were separated from their partner(s) before or during the asylum-seeking phase. Furthermore, the use of contraception depends on sexual activity and the desire to have a child.

In this study about a third of the women in the other African countries group had undergone FGM/C. The mean age at the time of FGM/C was five years. This and previous studies show that identifying women who have undergone FGM/C is important in Finland [37, 38]. FGM/C can cause gynaecological problems, severe menstrual pains, and complications during pregnancy and birth. Many women are unaware that these problems may be related to FGM/C [26]. Menstrual discomforts are not always related to FGM/C, and all women who come from countries where sexual education is limited or not available at all, may have uncertainties regarding issues related to menstruation [39].

### Reproductive health

Almost half of the women in the Russia and FSU group had had three or more pregnancies (including miscarriages and induced abortions) and a third had had at least one induced abortion. A quarter of the women from other African countries were pregnant at the time of study. Previous research conducted in Finland, which combined survey data and registry information, also indicates that women of Russian background often experience recurrent induced abortions. Under-reporting of induced abortions was observed among Somali origin women. Additionally, 64% of women of Somali background had at least three births [22]. Despite its frequency and availability in most western countries and as well as in Russia, abortion remains a highly sensitive, stigmatized, and difficult-to-measure experience [40].

In this study, 36–68% of women reported childbirth difficulties, including severe tears requiring medical interventions, C-sections or other procedures, and unusually painful or prolonged labor. Previous research indicates that women from diverse backgrounds and vulnerable positions experience more negative pregnancy and childbirth outcomes, as well as a higher incidence of pregnancy- and childbirth-related complications [41]. Although childbirth experiences are also subjective, our results highlight the need for discussions with women about these issues.

We observed menstrual problems to be quite common. Asylum-seeking women might encounter many difficulties in availability of female-friendly hygiene, sanitation facilities and menstrual hygiene products [42]. Insufficient hygiene can cause, for example, infections [14]. SRH literacy is likely to be low among refugee and asylum-seeking women. They may not have sufficient information about menstrual health and they may not recognize their own need for medical help [43]. A previous qualitative study shows that in some cultures there is a lot of shame associated with menstruation [28]. Syrian women interviewed in the study told that in their culture things related to menstruation should be kept hidden: talking about menstruation, menstrual products, menstrual pain and other symptoms isn’t acceptable. Women had received their menstrual-related information mainly from the female members of their social environment, e.g. mothers.

### Feasibility and acceptability of addressing questions of sexual and reproductive health

Refusal to participate in studies or unwillingness to answer to questions related to SHR can compromise both the internal and external validity of findings [16]. In our study, it was not possible to examine reporting bias with statistical methods or compare the results with other data, as there were no comparable previous studies or statistics on this topic and target group. Nonresponse was assessed by studying refusals to answer the questions concerning sexual health. Based on this, the acceptability of sensitive questions was evaluated. The overall percentage of women who skipped the question or did not wish to answer (i.e. nonresponse) varied between 7 and 17%. This can be considered acceptable for providing information on service needs, although not giving fully valid results on prevalence of problems related to SRH.

We did not have the opportunity to reliably investigate nonresponses to questions related to reproductive health. Before the TERTTU survey, some questions regarding SRH such as contraception and menstruation were not considered as sensitive as the questions on e.g. sexual behaviour. However, previous research shows that information regarding contraceptive use can also be unreliable. This is due to problems in recalling the contraceptive methods used and the acceptability of contraception in some cultures, which may lead to respondents not answering honestly or at all [40]. In the future the sensitivity of reproductive health issues (such as contraceptive use and menstrual health) needs more attention in choosing the questions and the way they are introduced.

The results of this study indicate that although questions related to sexual and reproductive health can be sensitive, and therefore e.g. questions about sexual activity are not always answered truthfully, asking about these topics is both acceptable and, most importantly, essential during the initial health examination of newly-arrived asylum seekers, but the sensitivity of the questions must be taken into account, especially when addressing topics that may be linked to shame and stigma.

In surveys, it is important to assess SRH using standardized methods tailored to the needs of asylum-seekers and to consider the sensitivity of the questions. In our study, the survey questionnaires were translated into different languages and interpreters were used when necessary, as suggested earlier [22].The reasons behind nonresponse may have to do with, for example, cultures in which these questions can be perceived particularly sensitive. Culturally sensitive survey protocols, such as ensuring that the research nurse is of the same sex as the research participant whenever possible, help reduce reporting bias [44]. This was not always possible in our study. The research participants had the opportunity to discuss the sensitive questions with research nurses before and after answering, as also suggested in a previous study [27]. It is important to explain and justify why questions related to SRH are being asked. In our study, the explanatory texts alongside the survey questions and the building of trust with the survey participants, starting from the first contacts with them, could have been the reason for a relatively low nonresponse.

### Health examinations and other health services

Comprehensive health examinations should be offered to asylum-seeking women soon after their arrival, as they are in a vulnerable situation [45, 46]. Our results support the aim of addressing SRH (sexual health, FGM/C, contraception, births, pregnancies, and menstrual health) during these examinations to provide individual information and support [26]. Discussing SRH can help identify women at risk, such as those who have experienced violence, enabling better guidance and counselling, and the design of services to meet their needs.

In Finland, pregnant asylum seekers are entitled to maternity care [47]. The regional health service system provides maternity clinic services to all residents, and authorities must guarantee access to public preventive health services for pregnant women [48], where professionals also need to identify the specific needs of asylum seeking women.

### Strengths and limitations

This study has several strengths. The sensitivity of the topic and the reliability of the information received from the participants were contemplated and taken into consideration in many ways in the TERTTU survey. The data collection was implemented by eight trained multilingual research nurses, and the survey material was translated into additional languages over the course of the survey when the need arose. Standardised data collection procedures were used in the interviews with the asylum-seeking women. The training of research nurses included information on ethical issues, introduction to research themes, and guidelines on how to ask sensitive questions. The research nurses’ work was organized in such a way that in most cases the nurse and the participant had a common language and the same sex.

The research nurses had the opportunity to assist with answering if needed. For example, they could explain why questions about SRH were being asked and what the different questions meant. Additionally, research nurses were instructed to try to ensure that the responses to questions concerning SRH were as comprehensive as possible.

The results of our study show that an examination by country of origin is important, as there are clear differences between the groups. A previous study utilizing the TERTTU survey data showed significant differences between the study groups in trauma experiences [49].

This study has also some limitations. The fact that some participants did not wish to answer all questions about SRH is a major limitation, as we do not know if these women had more or less risks for SHR problems than those who responded to all questions.

We aimed to assess non-responses to reproductive health questions, but there is so much uncertainty related to the lack of responses that we cannot report the results. It is unclear whether non-responses indicate a lack of reproductive health events or a reluctance to answer. The presence of an interpreter (among 39% of participants) may have affected women's willingness to answer and the validity of their answers.

In a separate study [50], the TERTTU research nurses were interviewed to assess the reliability of the information and to develop future research methodologies. They reported that SRH questions were difficult for some participants, especially when the interviewer and the interpreter were of the opposite sex. While independent questionnaire completion eased asking sensitive questions, nurses worried about blank responses [50]. However, no major gaps in obtained data or frequently missing items were observed in the data for our study.

The small size of the study population prevented reporting proportions of same-sex sexual activity, repeated miscarriages or induced abortions (≥ 3), and sexual and reproductive health outcomes across different socioeconomic groups. The question regarding miscarriages is difficult in terms of reliability. Especially for asylum-seeking women, it can be difficult to follow the menstrual cycle and notice early miscarriages, as they have often been in conditions where it can be difficult to track the regularity of their menstrual cycles. However, this question is crucial for identifying women who need reproductive health counselling and support.

Generalizing our findings on the prevalence of SRH problems among asylum seekers to the current context should be viewed with caution as the countries of birth as well as the reasons for leaving these countries as asylum seekers vary from year to year.

In the future, it is important to study differences in SRH between socioeconomic groups with a larger data set. Qualitative studies are also needed to reveal issues related to the sensitivity of the SRH questions and to develop fieldwork practices to reduce non-response. Furthermore, it is important to include asylum-seeking women’s voices in conversations about their sexual and reproductive health and rights [3].

## Conclusions

In this study, the study population represents the newly-arrived asylum seekers in Finland in 2018. A fifth of the women who participated in this study reported the need for contraception and a tenth of them were pregnant. Different SRH challenges were observed and found prevalent especially among women coming from other African countries and Russia and FSU. Based on the findings of our study, it feels necessary in the future to pay attention to service needs related to SRH among women throughout the asylum-seeking process. Even though some participants did not wish to answer all the questions on SRH, our study shows that it is feasible and useful to include SRH issues in the regular health examinations offered to all newly arrived asylum seekers in Finland. In addition, in the future gender and sexual diversity should be better taken into account when planning survey questionnaires. Based on the TERTTU survey experiences, these topics were also included in the manual for asylum seekers’ health examinations, to be applied in reception centres in Finland [48].

## Supplementary Information

Additional file1.

## Data Availability

The data that support the findings of this study can be obtained from THL but restrictions apply due to data protection regulations, ethics approval of the surveys and the subjects consent to participate. Thus the data are not publicly available.

## Abbreviations

Cl: Confidence intervals.
FGM/C: Female genital mutilation/cutting.
FSU: Russia or the former Soviet Union.
MENA: Middle East and North Africa.
NA: Not available.
SRH: Sexual and reproductive health.
