# Supplementary material for: Sexual and reproductive health of newly-arrived asylum-seeking women: a cross-sectional survey in Finland
Source: Reprod Health. 2025 Apr 26;22:59. doi: 10.1186/s12978-025-02012-2 (PMC12032761; doi:10.1186/s12978-025-02012-2)
Supplement: Supplementary file 1 — Additional file1. [file 12978_2025_2012_MOESM1_ESM.pdf]

Appendix 1. Variables

Theme	Question / Data source	Answer options	Categories
**Background information**			
Gender	Register information	-	-man<br>-woman
Country of birth	Register information	-	-Russia or the former Soviet Union<br>-the Middle East and North Africa<br>-other African countries (exl. North Africa)<br>-other countries (the most common countries of birth were Nicaragua, Albania, Bangladesh, India, Cuba, Kosovo, and Sri Lanka).
Age	Register information	-	-18–29 years<br>-30–39 years<br>-39–50 years
Education	*What is your highest completed education or degree?*	-Unknown<br>-No education<br>-Basic education 3 years or less<br>-Basic education 4 to 6 years<br>-Basic education 7 to 9 years<br>-Upper secondary education / High school<br>-Vocational school<br>-Tertiary education [bachelor's or master's degree or higher]	-No education or only elementary school education<br>-High school or vocational training<br>-University degree
Reading skills	*How well can you read?*	-I can read all kinds of text<br>-I can read simple text<br>-I can read names, words and very simple sentences<br>-I can not read at all	-Can at most read simple texts<br>-Can read all kind of texts
Writing skills	*How well can you write?*	-I can write all kinds of text -<br>-I can write simple text<br>-I can write familiar names, words and very simple sentences<br>-I can not write at all	-Can at most write simple texts<br>-Can write all kind of texts
Language skills	*Do you speak any other languages besides your mother tongue?*	-No<br>-Arabic<br>-Kurdish<br>-Dari<br>-Persian	-Speaks only her mother tongue<br>-Speaks several languages

<table>
<tr><td></td><td></td><td>-Somali
-Russian
-English
-French
-Other language, what?</td><td></td></tr>
<tr><td>Family</td><td>Do you have a spouse?</td><td>-No
-Dead
-In Finland
-Elsewhere, in [country/countries]</td><td>-No spouse
-Spouse in Finland or abroad</td></tr>
<tr><td colspan="4">Sexual health</td></tr>
<tr><td colspan="4">In the questionnaire, the questions about sexual health were introduced with a note: “This questionnaire has questions related to sexual and reproductive health. In Finland, it is forbidden to discriminate against anyone based on sexuality or sexual behaviour. With these questions we can identify risk behaviour related to diseases and recognize the need for care, protection, support and help. It is important that you answer these questions according to your situation without considering what is usually acceptable in your own society. Remember that the information you provide is handled with confidentially and the results are presented in a summarized form, from which individual respondents cannot be identified.”</td></tr>
<tr><td>Sexual activity</td><td>Have you had sexual intercourse during the past 12 months?</td><td>-No
-Yes
-I do not want to answer</td><td>-Yes
-No</td></tr>
<tr><td>The number of sexual partners</td><td>How many sexual partners have you had during the past 12 months?</td><td>-One
-2–5 partners
-6–10 partners
-More than 10 partners
-I do not want to answer</td><td>-More than one sex partner in past 12 months
-Six or more sex partners in past 12 months</td></tr>
<tr><td>The gender of the sexual partner(s)</td><td>During the past 12 months, have you had sex:</td><td>-With a man
-With a woman
-No sex during the past 12 months
-I do not wish/want to answer</td><td>-Partner opposite sex
-Partner same sex</td></tr>
<tr><td>Use of contraceptive</td><td>The next question is about contraceptive methods that protect from sexually transmitted diseases and unwanted pregnancies.
Did you use a contraceptive method during your most recent sexual intercourse?</td><td>-Only a condom
-A condom and another contraceptive method
-Not a condom, only another contraceptive method
-No contraception
-I do not wish to answer</td><td>-Did use contraceptive(s) during the latest intercourse
-Did not use any contraceptives during the latest intercourse</td></tr>
<tr><td>Need of contraceptive</td><td>Do you currently need contraceptive?</td><td>-Yes
-No</td><td>-Contraception need</td></tr>
<tr><td colspan="4">After the questions about the use of contraceptive methods, the respondents were given information on the topic: “Unprotected sexual intercourse increases the risk for sexually transmitted diseases. Using a condom effectively protects from these diseases. You can buy condoms at the grocery store or a pharmacy or ask for them from the nurse at your reception centre. Other methods of contraception are also available to prevent unwanted pregnancies.”</td></tr>
</table>

The following introduction was given before questions about FGM/C: “Female circumcision is a procedure where female external genital organs are partially or completely removed or harmed in another way based on cultural or other non-medical reasons.”			
Female genital mutilation / cutting	*In some countries, girls or boys are circumcised, which can have an impact on health.*<br>*Have you been circumcised?*	-Yes<br>-No<br>If yes, the respondent was asked to specify at what age they had been circumcised.	-Yes<br>-No
**Reproductive health**			
Pregnancy	*Are you pregnant?*<br><br>[If the woman answered yes, pregnancy weeks were either recorded or marked unknown. When necessary, the research nurses were instructed to help to estimate the pregnancy weeks.]	-Yes<br>-No<br>-I do not know	-Yes<br>-No
Previous pregnancies	*How many pregnancies have you had?*	-The number was recorded (0 if none)	-0<br>-1–2<br>-3 or more
Previous births	*How many births have you had?*	-The number was recorded (0 if none)	-0<br>-1–2<br>-3 or more
Previous miscarriages	*How many miscarriages have you had?*	-The number was recorded (0 if none)	-0<br>-1–2<br>-3 or more
Previous abortions	*How many abortions have you had?*	-The number was recorded (0 if none)	-0<br>-1–2<br>-3 or more
Problems related to child births and menstrual health	*Are you experiencing/have you experienced the following women’s health problems?*<br>*This means lately or during latest period/birth:*<br>*-Disruptive pain during menstruation (e.g. pain so difficult that you are unable to deal with everyday tasks or need strong pain killers)*<br>*-Heavy menstruation (e.g. a heavy flow that disturbs everyday tasks or lasts longer than normal)*<br>*-Irregular menstruation (e.g. irregular cycle or beginning of flow is difficult to estimate)*	-No<br>-Yes	-Yes<br>-No

	-Difficulties during labour (e.g. a difficult tear stitched by a doctor/stitched under sedation), caesarean section or another procedure, labor more painful than normal or prolonged labour)		
